# High plasma nesfatin-1 level in Chinese adolescents with depression

**DOI:** 10.1038/s41598-023-42513-3

**Published:** 2023-09-15

**Authors:** Jin Sun, Nannan Gao, Qiong Wu, Yan Li, Li Zhang, Zhongliang Jiang, Zhiyi Wang, Jintong Liu

**Affiliations:** 1 Department of Pediatrics, The First Affiliated Hospital of Shandong First Medical University & Shandong Province Qianfoshan Hospital, Shandong Engineering and Technology Research Center for Pediatric Drug Development, Jinan, China; 2 Key Laboratory of Birth Regulation and Control Technology of National Health Commission of China, Maternal and Child Health Care Hospital of Shandong Province Affiliated to Qingdao University, Jinan, China; 3 Present Address: Department of Psychiatry, Shandong Mental Health Center, Shandong University, Jinan, China

**Keywords:** Psychology, Biomarkers, Medical research

## Abstract

Depression is a common psychiatric disorder with high prevalence and mortality rates as well as high risk of serious harm in adolescents that have significant negative impact on families and society. The feeding inhibitor Nesfatin-1 contributes to the regulation of stress and emotion. The purpose of this project was to compare the differences in the levels of Nesfatin-1 between adolescents with depression and healthy adolescents, and verify the association between the levels of Nesfatin-1 and severity of depression in adolescents. Adolescents with depression (n = 61) and healthy adolescents (n = 30) were evaluated. The Hamilton Depression Rating Scale (HAMD-17) was used to classify the adolescents with depression. Thirty-one and thirty-two was assigned to the mild-to-moderate (HAMD-17 ≤ 24) depression group and severe group (HAMD-17 > 24). Plasma Levels of Nesfatin-1 were measured by human ELISA Kit and differences among groups evaluated. Data were analyzed using the statistical software SPSS 23. HAMD-17 score was significantly higher in adolescents with depression than that in the healthy adolescents (P < 0.001). Median plasma Nesfatin-1 levels in adolescents with depression and healthy adolescents differed significantly at 37.3 pg/ml (22.1 pg/ml, 63.6 pg/ml) and 18.1 pg/ml (10.0 pg/ml, 25.7 pg/ml) (p < 0.001). A multivariate logistic regression analysis showed high plasma Nesfatin-1 concentrations were associated with increased risk of depression (OR = 0.914, 95% CI 0.87–0.96, P < 0.001). The receiver operating characteristic curve showed that the area under curve were 0.808 (95% CI 0.722–0.894, P < 0.001). Plasma Nesfatin-1 cut-off point of 32.45 pg/mL showed 59% sensitivity and 100% specificity. Median plasma Nesfatin-1 levels in the severe depression group (n = 30), mild-to-moderate depression group (n = 31), and control group (n = 30) were 53.4 pg/ml (28.2 pg/ml, 149.1 pg/ml), 29.9 pg/ml (14.5 pg/ml, 48.5 pg/ml) and 18.1 pg/ml (10.0 pg/ml, 25.7 pg/ml), and differed significantly among the three groups (P < 0.001). Median plasma level of Nesfatin-1 in males (n = 20) was 38.6 pg/ml (23.5 pg/ml, 70.1 pg/ml), while that in females (n = 41) was 37.3 pg/ml (22.0 pg/ml, 63.6 pg/ml), which was not a significant difference (P > 0.05). Plasma levels of Nesfatin-1 increased with severity of depression in adolescents and may be useful as a biomarker of depression severity. Further studies are needed in future projects.

## Introduction

As a common clinical mental disorder, depression is mainly manifested as significant and continuous disappointment and sadness, as well as loss of interest enjoyable or rewarding activities. Depression can also disrupt sleep and appetite. Approximately 280 million people have depression worldwide^1^. According to statistics from the World Health Organization, depression will rise to become the largest global disease burden by 2030^2^.

Globally, mental health disorders account for 16% of the burden of disease and injury in people among 13–18 years old^2^. Half of all mental health disorders in adulthood start before age 14, but most cases were not diagnosed and appreciated, which increase the risk of affective disorders and other mental illnesses in adulthood^3^. The adverse consequences will extend into adulthood if mental health problems were not addressed, impairing both physical and mental health^4^. The prevalence of depression in adolescents in the U.S. is 11.0%, with a 1-year prevalence of 7.5%^5^. According to the results of the China mental health survey in 2019, the prevalence rate of depression in China is 3.4%, and the 12-month prevalence rate is 2.1%^6^.

Depression is an important cause of suicide among adolescents, while suicide brings heavy burdens to entire family and society^7^. With the rising incidence of depression among adolescents, the suicide rate of middle school students also rises year by year^8^. Stressful life events, poor family environment, cruel sadness, excessive use of the internet lead to an increase of psychiatric disorders in adolescents, including anxiety and depression^9^. Globally, adolescents in different countries have experienced higher levels of stress, anxiety, and depression due to the pandemic^10^. Adolescence is a period of physical and mental development, and the high prevalence, serious harm, and high mortality rate associated with adolescent depression have huge impacts on adolescents themselves, their families, and society^8^. It is necessary for more academics and physicians in related specialties to research on adolescent mental disorders which will lead to an increase in psychiatric illness in adulthood. There are currently no objective biomarkers to aid early recognition, diagnosis, and prognosis evaluation of depression in adolescents.

Nesfatin-1 is a feeding inhibitor, an 82-amino acid polypeptide hydrolyzed by nucleobindin-2 (NUCB2), was originally identified by Oh-I and colleagues in 2006^11^. Nesfatin-1 is broadly distributed in both the central nervous system and peripheral system^12^, indicating that it has a wide range of physiological effects. Nesfatin-1 play important roles in regulation of food intake, glucose homeostasis, and cardiovascular and reproductive functions^13,14^, and recent data show that it also contributes to the regulation of stress and emotion^15^, supporting its involvement in psychiatric disorders. Patients with depression almost always have accompanying symptoms of anorexia and metabolic abnormalities. However, the exact impact of Nesfatin-1 on depression and its potential mechanisms are still unclear. The pathogenesis may be related to stress and subsequent hyperactivity of the hypothalamic–pituitary–adrenal (HPA) axis^16^, and similar mechanisms may be associated with dysfunction of the HPA and hypothalamic-pituitary-thyroid axes^17^. Altered regulation of adrenocorticotropic hormone and cortisol secretion, as well as impaired corticosteroid receptor signaling, are thought to underlie depressive psychopathology^18^.

Several studies have demonstrated that plasma Nesfatin-1 levels are clearly increased in adults with depression relative to healthy subjects and that there is a positive correlation between Nesfatin-1 levels and the severity of depression^19–21^. One study found that the serum nesfatin-1 levels were significantly lower in the major depressive disorder group than the control group, and did not find a correlation between the severity of depression and the Nesfatin-1 levels in adolescents with depression, which conclusion was different from other studies^22^. Moreover, earlier identification and treatment of individuals with depression would be beneficial. Previous studies of depression have focused primarily on middle-aged and older people. Burak’ study investigated a positive correlation between serum Nesfatin-1 levels and CDI scores in adolescents diagnosed as major depressive disorder^23^. The aim of our study was to compare the differences in the levels of Nesfatin-1 between adolescents with depression and healthy adolescents, and verify the association between the levels of nesfatin-1 and severity of depression in adolescents.

## Materials and methods

### Participants

This experimental subject was researched and approved by the Medical Ethics Committee of the Shandong Mental Health Center. The purpose of this project was explained to all participants and their parents, with informed consent was obtained.

Research participants were selected from the Child and Adolescent Mental Health Department, Shandong Mental Health Center. Obese individuals were excluded when selecting patients and control groups. Sixty-one adolescents were diagnosed with MDD from November 2020 to December 2021. The inclusion criteria for the patient group were: (a) age 13–18 years; (b) diagnosed with depression using the fifth version of Diagnostic and Statistical Manual for Psychiatric Disorders (DSM-5) criteria; (c) score on the HAMD-17 > 7^24^.

The control group were selected from individuals undergoing health examination at Shandong Mental Health Center from November 2020 to December 2021. The inclusion criteria for the control group were: (a) age 13–18 years and (b) no mental disorder after assessment by psychiatrists. The exclusion criteria for patients and controls were as follows: physical diseases, including cardiac, respiratory, renal, or endocrine diseases; or receiving any hormonal or drug therapy.

Height and weight of all participants were precisely measured and recorded, and body mass index (BMI) calculated as weight/height^2^ (kg/m^2^). Depression severity was classified using HAMD-17, as follows: 0 ≤ score ≤ 7, no depression; 8 ≤ score ≤ 17, mild depression; 18 ≤ score ≤ 24, moderate depression; score ≥ 25, severe depression^24^. The study was conducted in accordance with the Declaration of Helsinki.

### Laboratory measurements

Blood samples (3–5 ml) were collected in tubes containing EDTA at 8:00 am after an overnight fasting period, immediately put it into a low temperature centrifuge for centrifugation (Biofuge Stratos, Thermo Fisher Scientific, America, 3000×*g* for 10 min at 4 °C), and plasma were aspirated and stored at − 80 °C until further processing. Blood was also collected to test liver and kidney function, biochemistry, thyroid function, glucose, and sex hormones, to exclude influencing factors and related diseases. Plasma Nesfatin-1 level was measured using the Human Nesfatin-1 Double Antibody Sandwich ELISA Kit (Boster Biological Technology, Wuhan, China. No. EK1138).

### Statistical analysis

Data were analyzed using the statistical software SPSS 23. Continuous variables were examined using the Kolmogorov–Smirnov test to determine whether or not they were normally distributed. Data were described as mean ± SD or median (quartiles). Categorical variables were described by the number of cases (percentages). Comparisons between two groups were performed using T test or Mann–Whitney U-test. Comparisons between multiple groups were performed using ANOVA or Kruskal–Wallis-test, the intra-group comparisons were made using the calibration alpha method. A multivariate logistic regression analysis was performed to validate the risk factors of depression. The receiver operating characteristic (ROC) curve analysis was used to determine the cut-off value of plasma Nesfatin-1. Results were considered statistically significant at p < 0.05.

### Ethical approval and consent to participate

This experimental subject was researched and approved by the Medical Ethics Committee of the Shandong Mental Health Center. Written informed consent to participate in this study was provided by the participants and the participants’ legal guardian/next of kin, if participants were under 18 years old.

## Results

### Participant characteristics

The demographic characteristics of the patient and control groups are presented in Table 1. There were no significant differences in age, sex, glucose, or BMI between groups (p > 0.05). Mean HAMD-17 score was significantly higher in adolescents with depression than that in the control group (p < 0.001).

**Table 1:** Comparisons of the experimental and control groups.

	Depression group (n = 61)	Control group (n = 30)	*P* value
Age (years)	14.7 ± 1.4	14.3 ± 1.0	0.076
Male, n (%)	20(32.8)	14(46.7)	0.198^a^
BMI (kg/m^2^)	20.5 ± 3.5	19.8 ± 3.6	0.376
Glucose (mmol/L)	4.89 ± 0.47	4.91 ± 0.5	0.821
HAMD-17	24.9 ± 7.4	3.3 ± 1.5	< 0.001
Nesfatin-1 level (pg/ml), median (P25, P75)	37.3 (22.1, 63.6)	18.1 (10.0, 25.7)	< 0.001^b^

*BMI* body mass index, *HAMD* Hamilton depression rating scale, *P25* 25th percentile, *P75* 75th percentile.

^a^Calculated by Chi-squared test.

^b^Calculated by Mann–Whitney U-test.

Median plasma level of Nesfatin-1 in adolescents with depression was 37.3 pg/ml (22.1 pg/ml, 63.6 pg/ml), while that in healthy adolescents was 18.1 pg/ml (10.0 pg/ml, 25.7 pg/ml) (Table 1); hence, adolescents with depression had significantly higher Nesfatin-1 levels than healthy adolescents (p < 0.001).

A multivariate logistic regression analysis was constructed by age, sex, BMI and plasma Nesfatin-1 level (Table 2). Depression was set as a dependent variable, other indicators were set as independent variables. Older age (OR = 0.609, 95% CI 0.393–0.944, P < 0.05), plasma Nesfatin-1 (OR = 0.914, 95% CI 0.87–0.96, P < 0.001) were the independent indicators for adolescents with depression.

**Table 2:** Multivariate logistic regression analysis constructed by age, sex, BMI and nesfatin-1 level.

	β (95%confidence interval)	SE	*P* value
Age	0.609 (0.393–0.944)	0.224	0.027
Sex	2.594 (0.823–8.177)	0.586	0.104
BMI	0.92 (0.781–1.083)	0.083	0.318
Nesfatin-1 level	0.914 (0.87–0.96)	0.025	< 0.001

*BMI* body mass index.

**Figure 1:**
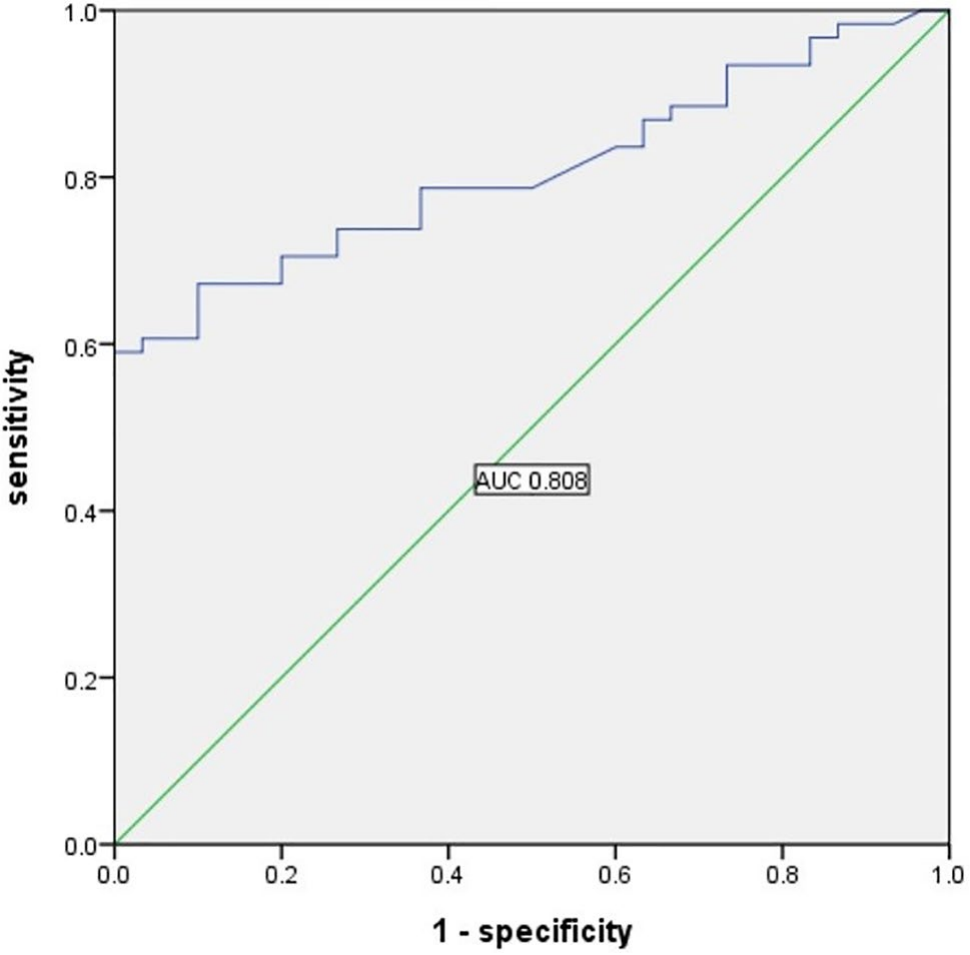
ROC curve of plasma nesfatin-1 in identification of the adolescents with depression.

The ROC curve showed that the area under curve (AUC) were 0.808 (95% CI 0.722–0.894, P < 0.001). Plasma Nesfatin-1 cut-off point of 32.45 pg/mL showed 59% sensitivity and 100% specificity (Fig. 1).

**Figure 2:**
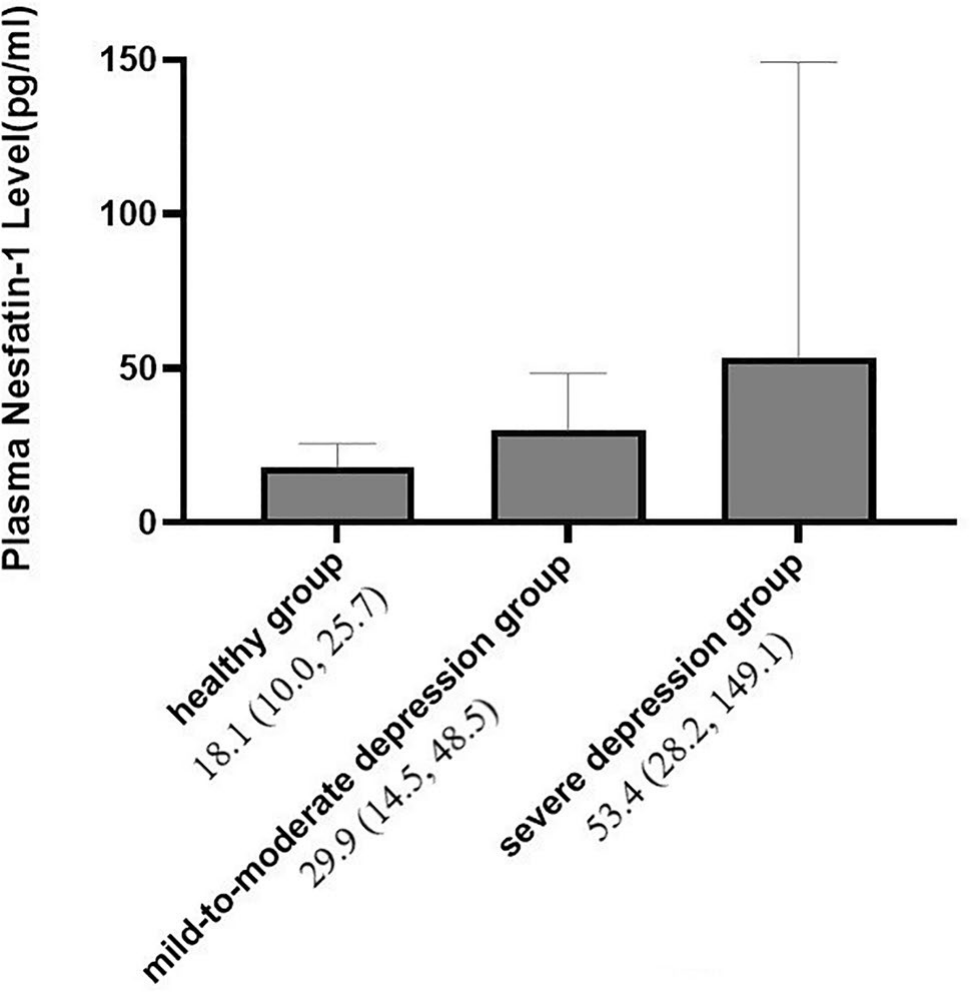
Plasma nesfatin-1 levels in the healthy group, the mild-to-moderate depression group, and the severe depression group.

Among the sixty-one adolescents in the patient group, thirty-one had mild-to-moderate depression and thirty had severe depression, while the control group comprised thirty healthy adolescents. Median plasma Nesfatin-1 levels in the severe depression group, mild-to-moderate depression group, and control group were 53.4 pg/ml (28.2 pg/ml, 149.1 pg/ml), 29.9 pg/ml (14.5 pg/ml, 48.5 pg/ml), and 18.1 pg/ml (10.0 pg/ml, 25.7 pg/ml), respectively (Table 3), and there was a significant difference in Nesfatin-1 levels among the three groups (H = 29.3, P < 0.001). Further, there was a significant difference in Nesfatin-1 level between the severe and mild-to-moderate depression groups (P = 0.006), the mild-to-moderate depression and healthy groups (P = 0.003), and the severe depression and healthy groups (P < 0.001). Figure 2 showed that a significant increased trend of the plasma Nesfatin-1 levels among the three groups.

**Table 3:** Plasma nesfatin-1 levels in severe depression group, mild-to-moderate depression group and control group.

Group	N	HAMD-17	Nesfatin-1 level (pg/ml), median (P25, P75)	P
Severe depression group	30	30.6 ± 5.4	53.4 (28.2, 149.1)	P < 0.05*
Mild-to-moderate depression group	31	19.5 ± 4.2	29.9 (14.5, 48.5)*	P < 0.05^+^
Control	30	3.3 ± 1.5	18.1 (10.0, 25.7)^+#^	P < 0.001^#^

*Compared severe depression group with mild-to-moderate depression group.

^+^Compared mild-to-moderate depression group with control group.

^#^Compared severe depression group with control group.

*^+#^Calculated by Kruskal–Wallis-test.

Median plasma level of Nesfatin-1 in males (n = 20) was 38.6 pg/ml (23.5 pg/ml, 70.1 pg/ml), while that in females (n = 41) was 37.3 pg/ml (22.0 pg/ml, 63.6 pg/ml) (Table 4), which was not a significant difference (P > 0.05).

**Table 4:** Plasma nesfatin-1 levels in adolescents with depression stratified by sex.

Group	M (P25, P75)	*P* value
Males (N = 20)	38.6 (23.5, 70.1)	0.89*
Females (N = 41)	37.3 (22.0, 63.6)	

*Calculated by Mann–Whitney U-test.

## Discussion

Nesfatin-1 levels are significantly higher in adults with major depressive disorder than in those with moderate depression or controls^21^. Plasma Nesfatin-1 levels in Chinese adults with depression were higher than healthy subjects, and they were positively correlated with the severity of depression^20^; hence, several previous studies have shown that plasma Nesfatin-1 level could be a potential indicator of depression severity. In the present study, we found higher plasma Nesfatin-1 levels in adolescents with depression relative to healthy controls, which result is contrary to Karadeniz’s research^22^. Further, higher Nesfatin-1 levels were correlated with increased depression severity, consistent with previous reports^19,20^. Nesfatin-1 may have important roles in the neuroendocrine regulation of stress^25^. The increased level of Nesfatin-1 was also consistent with previous studies, stress could also increase the plasma Nesfatin-1 levels^26^.

Nesfatin-1 has received considerable attention over the past 15 years, due to its effect of reducing food intake by influencing gut motility and feeding behavior^27,28^. Bloem and his team provided evidence of expression of Nesfatin-1 in the human depression^29^. Recent evidence also supports a relationship between depression and thyroid function^30–32^, and Nesfatin-1 levels are higher in hyperthyroid patients than controls^33^. Xu et al. reported that Nesfatin-1 levels were significantly higher in depressed patients with subclinical hypothyroidism than in healthy controls^17^. In this study, we excluded potential participants with medical conditions, including hypothyroidism or diabetes, when indicated by medical history and physical examination.

Further, the global prevalence of depression has been increasing in recent years, the rate of increase in adolescents exceeds that of adults^34^. Major Depressive Disorder is a major risk factor for suicide^35^. There was a negative correlation between suicidal ideation scores and Nesfatin-1 levels in depressed patients with suicidal ideation^36^. A blood marker to predict and monitor patient risk of depression and degree of depression severity would be of considerable value, and plasma Nesfatin-1 level is a potential biomarker of patients with severe depression.

Research suggests that risk factors for depression in adolescents include female gender, personal history of trauma, family history of mental illness, chronic disease, and family conflict^37^. Among adolescents, girls have higher overall rates of depression and more severe depressive symptoms than boys; moreover, the prevalence rates and severity are higher in adolescents with increasing age^5^. The present study included both females and males and, although Nesfatin-1 expression is reported to differ according to sex^38,39^, we found no significant difference in Nesfatin-1 levels between genders; this difference between our results and previous reports was likely related to variations in participant’s races, ages and sample sizes. This subject warrant thorough further investigation.

Several recent studies have provided evidence suggesting Nesfatin-1 involvement in other important brain functions, such as sleep, emotion regulation, anxiety, and depression^40^. Patients who suffer from sleep disorders often present with mental illnesses, such as depression^41^. The feeding inhibitory molecule Nesfatin-1 was recently identified as a potential mood regulator; and impaired appetite and altered metabolism are common in depression^42,43^; however, its precise effect on depression and the possible underlying mechanisms remains unclear. In Burak’ study, there is a positive correlation between serum nesfatin-1 levels and CDI scores in adolescents with depression. But the sample size is small, and CDI is vulnerable to certain limitations like other self-report assessments used in children and adolescents, because their responses may not reflect their true emotional state^23^. HAMD-17 is the most commonly used scale for assessing depression in clinical practice. In our study, we used the Chinese version. The total score can better reflect the severity of the disease. The higher the total score, the more severe the depression. The reliability and validity of this scale are good, with reliability and validity coefficients of 0.99 and 0.37^44^.

According to our research, this is one of the very few studies to assess whether plasma Nesfatin-1 levels have potential for application in predicting and appraising depression and its severity in adolescents. However, our study also has limitations, including the relatively small sample size; hence, the findings should be validated in a study with a larger sample size. In addition, future studies should incorporate more relevant factors to further clarify the underlying mechanism and identify correlations. In future studies, we will focus on further investigation of the correlation of Nesfatin-1 levels with thyroid function, blood sugar, glycosylated hemoglobin, cortisol, corticosterone, and other indicators in patients with depression. And we should further study the correlation between the level of Nesfatin-1 and the use of antidepressants.

## Conclusion

In conclusion, plasma Nesfatin-1 levels were significantly higher in adolescent patients with depression than those in healthy controls. Plasma levels of Nesfatin-1 increased with severity of depression in adolescents and may be useful as a biomarker for the diagnosis and assessment of depression. Longitudinal and mechanistic studies should be continued in our future projects.

## Data Availability

The data and materials during the study are available from the first author on reasonable request.

## References

[1] The Institute of Health Metrics and Evaluation. Global Health Data Exchange (GHDx). http://ghdx.healthdata.org/gbd-results-tool?params=gbd-api-2019-permalink/d780dffbe8a381b25e1416884959e88b. Accessed May 1, 2021.

[2] Rehm J, Shield KD (2019). Global burden of disease and the impact of mental and addictive disorders. Curr. Psychiatry Rep..

[3] World Health Organization (WHO). https://www.who.int/news-room/fact-sheets/detail/adolescents-health-risks-and-solutions.Assessed on 28/04/2023.

[4] Cullen KR, Gee DG, Klimes-Dougan B, Gabbay V, Hulvershorn L, Mueller BA (2009). A preliminary study of functional connectivity in comorbid adolescent depression. Neurosci. Lett..

[5] Avenevoli S, Swendsen J, He JP, Burstein M, Merikangas KR (2015). Major depression in the national comorbidity survey-adolescent supplement: Prevalence, correlates, and treatment. J. Am. Acad. Child Adolesc. Psychiatry.

[6] Huang Y, Wang Y, Wang H, Liu Z, Yu X, Yan J (2019). Prevalence of mental disorders in China: A cross-sectional epidemiological study. Lancet Psychiatry.

[7] Curtin SC (2020). State suicide rates among adolescents and young adults aged 10–24: United States, 2000–2018. Natl. Vital. Stat. Rep..

[8] Miller L, Campo JV (2021). Depression in adolescents. N. Engl. J. Med..

[9] Guessoum SB, Lachal J, Radjack R, Carretier E, Minassian S, Benoit L, Moro MR (2020). Adolescent psychiatric disorders during the COVID-19 pandemic and lockdown. Psychiatry Res..

[10] Jones EAK, Mitra AK, Bhuiyan AR (2021). Impact of COVID-19 on mental health in adolescents: A systematic review. Int. J. Environ. Res. Public Health.

[11] Oh-I S, Shimizu H, Satoh T, Okada S, Adachi S, Inoue K (2006). Identification of nesfatin-1 as a satiety molecule in the hypothalamus. Nature.

[12] Goebel-Stengel M, Wang L (2013). Central and peripheral expression and distribution of NUCB2/nesfatin-1. Curr. Pharm. Des..

[13] Aydin S (2013). Role of NUCB2/nesfatin-1 as a possible biomarker. Curr. Pharm. Des..

[14] Çelik F, Belviranli M, Okudan N (2016). Circulating levels of leptin, nesfatin-1 and kisspeptin in postmenopausal obese women. Arch. Physiol. Biochem..

[15] Wei Y, Li J, Wang H, Wang G (2018). NUCB2/nesfatin-1: Expression and functions in the regulation of emotion and stress. Prog. Neuropsychopharmacol. Biol. Psychiatry.

[16] Swaab DF, Bao AM, Lucassen PJ (2005). The stress system in the human brain in depression and neurodegeneration. Ageing Res. Rev..

[17] Xu YY, Liang J, Cao Y, Shan F, Liu Y, Xia QR (2017). High levels of Nesfatin-1 in relation to the dysfunction of the hypothalamic-pituitary-adrenal and hypothalamus-pituitary-thyroid axes in depressed patients with subclinical hypothyroidism. Neuropsychiatr. Dis. Treat..

[18] Lightman SL, Birnie MT, Conway-Campbell BL (2020). Dynamics of ACTH and cortisol secretion and implications for disease. Endocr. Rev..

[19] Ari M, Ozturk OH, Bez Y, Oktar S, Erduran D (2011). High plasma nesfatin-1 level in patients with major depressive disorder. Prog. Neuropsychopharmacol. Biol. Psychiatry.

[20] Xiao MM, Li JB, Jiang LL, Shao H, Wang BL (2018). Plasma nesfatin-1 level is associated with severity of depression in Chinese depressive patients. BMC Psychiatry.

[21] Algul S, Ozcelik O (2018). Evaluating the levels of nesfatin-1 and ghrelin hormones in patients with moderate and severe major depressive disorders. Psychiatry Investig..

[22] Karadeniz S, Yaman H, Bilginer Ç, Hızarcı Bulut S, Yaman SÖ (2020). Serum nesfatin-1, ghrelin, and lipid levels in adolescents with first episode drug naïve unipolar depression. Nord. J. Psychiatry..

[23] Burak Acikel S, Hosoglu E, Artik A, Humeyra Yerlikaya Aydemir F (2021). Increased serum nesfatin-1 levels among adolescents diagnosed with major depressive disorder. Arch. Clin. Psychiatry..

[24] Hamilton M (1960). A rating scale for depression. J. Neurol. Neurosurg. Psychiatry.

[25] Yoshida N, Maejima Y, Sedbazar U, Ando A, Kurita H, Damdindorj B (2010). Stressor-responsive central nesfatin-1 activates corticotropin-releasing hormone, noradrenaline and serotonin neurons and evokes hypothalamic-pituitary-adrenal axis. Aging (Albany NY).

[26] Xu YY, Ge JF, Qin G, Peng YN, Zhang CF, Liu XR (2015). Acute, but not chronic, stress increased the plasma concentration and hypothalamic mRNA expression of NUCB2/nesfatin-1 in rats. Neuropeptides.

[27] Stengel A, Taché Y (2010). Nesfatin-1–role as possible new potent regulator of food intake. Regul. Pept..

[28] Atsuchi K, Asakawa A, Ushikai M, Ataka K, Tsai M, Koyama K (2010). Centrally administered nesfatin-1 inhibits feeding behaviour and gastroduodenal motility in mice. NeuroReport.

[29] Bloem B, Xu L, Morava É (2012). Sex-specific differences in the dynamics of cocaine-and amphetamine-regulated transcript and nesfatin-1 expressions in the midbrain of suicide victims vs controls. Neuropharmacology.

[30] Almeida OP, Alfonso H, Flicker L, Hankey G, Chubb SA, Yeap BB (2011). Thyroid hormones and depression: The Health in Men study. Am. J. Geriatr. Psychiatry.

[31] Ojha SP, Dhungana S, Chapagain M, Tulachan P (2013). Association of thyroid dysfunction with depression in a teaching hospital. J. Nepal Health Res. Council.

[32] Liu F, Yang Q, Gao N, Liu F, Chen S (2014). Decreased plasma nesfatin-1 level is related to the thyroid dysfunction in patients with type 2 diabetes mellitus. J. Diabetes Res..

[33] Tohma Y, Akturk M, Altinova A, Yassibas M, Turgay E, Gulbahar O (2015). Circulating levels of orexin-A, nesfatin-1, agouti-related peptide, and neuropeptide Y in patients with hyperthyroidism. Thyroid.

[34] Weinberger AH, Gbedemah M, Martinez AM, Nash D, Galea S, Goodwin RD (2018). Trends in depression prevalence in the USA from 2005 to 2015: Widening disparities in vulnerable groups. Psychol. Med..

[35] Shooshtari MH, Malakouti SK, Panaghi L, Mohseni S, Mansouri N, Movaghar AR (2016). Factors associated with suicidal attempts in Iran: A systematic review. Iran J. Psychiatry Behav. Sci..

[36] Korucu CÇ, Atay İM, Zayıf SS, Gültekin F (2018). May nesfatin-1 be a state marker in major depressive disorder with suicidal ideation?. Psychiatry Res..

[37] Siu AL (2016). Screening for depression in children and adolescents: US Preventive Services Task Force recommendation statement. Ann. Intern. Med..

[38] Xu L, Bloem B, Gaszner B, Roubos EW, Kozicz T (2009). Sex-specific effects of fasting on urocortin 1, cocaine-and amphetamine-regulated transcript peptide and nesfatin-1 expression in the rat Edinger-Westphal nucleus. Neuroscience.

[39] Hofmann T, Elbelt U, Ahnis A, Rose M, Klapp BF, Stengel A (2015). Sex-specific regulation of NUCB2/nesfatin-1: Differential implication in anxiety in obese men and women. Psychoneuroendocrinology.

[40] Könczöl K, Bodnár I, Zelena D, Pintér O, Sugárka R, Palkovits M (2010). Nesfatin-1/NUCB2 may participate in the activation of the hypothalamic-pituitary-adrenal axis in rats. Neurochem. Int..

[41] Vas S, Ádori C, Könczöl K, Kátai Z, Pap D, Papp R (2013). Nesfatin-1/NUCB2 as a potential new element of sleep regulation in rats. PLoS ONE.

[42] Baxter LC (2016). Appetite changes in depression. Am. J. Psychiatry.

[43] Ghosh A, Dinakaran D, Nebhinani N, Andrade C (2017). Association between depression and metabolic syndrome: Critical issues and missed opportunities. Indian J. Psychiatry.

[44] Tang YH, Zhang MY (1984). Hamilton depression scale (HAMD). Shanghai Arch. Psychiatry.

